# To Obtund Sensative Dentin

**Published:** 1900-04-15

**Authors:** 


To Obtund Sensative Dentin.—Dip a pledget of cotton into strong solution of carbolic acid and then into powdered cocain, warm and place in the cavity. This will obtund sufficiently in a few minutes to make it practicable to apply granulated chloride of zinc without pain. In one and a half minutes the dentin will be thoroughly obtunded.—*Dr. Bogue, Dental Brief.*

